# Impairments in learning and memory performances associated with nicotinic receptor expression in the honeybee *Apis mellifera* after exposure to a sublethal dose of sulfoxaflor

**DOI:** 10.1371/journal.pone.0272514

**Published:** 2022-08-03

**Authors:** Alison Cartereau, Xavier Pineau, Jacques Lebreton, Monique Mathé-Allainmat, Emiliane Taillebois, Steeve H. Thany

**Affiliations:** 1 Laboratoire de Biologie des Ligneux et des Grandes Cultures (LBLGC) USC INRAE 1328, Université d’Orléans, Orléans, France; 2 CEISAM UMR CNRS 6230, UFR des Sciences et des Techniques, Université de Nantes, Nantes, France; Institut Sophia Agrobiotech, FRANCE

## Abstract

Sulfoxaflor is a new insecticide which acts on the nicotinic acetylcholine receptor (nAChRs) in a similar way to neonicotinoids. However, sufloxaflor (SFX) is thought to act in a different manner and is thus proposed as an alternative in crop protection. The goal of this study is to evaluate the toxicity of SFX and its sublethal effect on the honeybee *Apis mellifera* after acute exposure. In toxicological assay studies, the LD_50_ value and sublethal dose (corresponding to the NOEL: no observed effect level) were 96 and 15 ng/bee, respectively. Using the proboscis extension response paradigm, we found that an SFX dose of 15 ng/bee significantly impairs learning and memory retrieval when applied 12 h before conditioning or 24 h after olfactory conditioning. SFX had no effect on honeybee olfactory performance when exposure happened after the conditioning. Relative quantitative PCR experiments performed on the six nicotinic acetylcholine receptor subunits demonstrated that they are differently expressed in the honeybee brain after SFX exposure, whether before or after conditioning. We found that intoxicated bees with learning defects showed a strong expression of the Amelβ1 subunit. They displayed overexpression of Amelα9 and Amelβ2, and down-regulation of Amelα1, Amelα3 and Amelα7 subunits. These results demonstrated for the first time that a sublethal dose of SFX could affect honeybee learning and memory performance and modulate the expression of specific nAChR subunits in the brain.

## Introduction

Honeybees are major pollinators for crops and plants, and are consequently essential for the agricultural economy. Several studies have described pesticide exposure as one of the major threats to colony survival [1]. In particular, neonicotinoid insecticides, which are widely used to control a broad range of insect pests [2], are largely to blame for the decline of honeybee colonies [3, 4]. Exposure of honeybees to sublethal doses of neonicotinoid insecticides has led to behavioral and physiological impairments [5]. For example, low doses of clothianidin and imidacloprid affect honeybee foraging abilities [3], resulting in a significant decrease in colony survival [6–8]. Moreover, a recent study showed that neonicotinoids have compound-specific effects on the ability of bees to perform a complex olfactory learning task [9, 10]. Three neonicotinoids, clothianidin (CLT), imidacloprid (IMI) and thiamethoxam (TMX) were banned by the European Union in 2013 due to their adverse effects on honeybees [11].

The adverse effects of neonicotinoids on the honeybee have highlighted the importance of developing new compounds that are efficient against insect pests and safer for non-target species such as pollinators. Sulfoxaflor (SFX) is a relatively new compound belonging to sulfoximine insecticides [12] and has been marketed as being able to replace neonicotinoid insecticides [13]. SFX demonstrated high levels of insecticidal potency against sap-feeding insect species such as the sweetpotato whitefly, *Bemisia tabaci*, and the brown planthopper *Nilaparvata lugens*, which have developed resistance to IMI [13]. SFX’s effect was studied by the International Organization for biological Control (IOBC), and revealed a low impact on beneficial arthropods such as *Macrolophus caliginosus* (Hemiptera), and *Harmonia axyridis* (Coleoptera) [14]. It was recently shown that exposure of solitary bees to a field-realistic dose of SFX leads to impairment of foraging and flight performances [15]. Other authors described no major impacts of SFX on honeybees in a realistic-exposure protocol [16].

Unfortunately, no studies have evaluated the effect of a sublethal dose of SFX in relation to the expression of nicotinic acetylcholine receptor (nAChR) subunits in the brain. Indeed, SFX is classified as a neuronal nAChR competitive modulator by the Insecticide Resistance Action Committee (IRAC). SFX acts on nicotinic acetylcholine receptors (nAChRs) present in the central nervous system of insects in a similar way to neonicotinoid insecticides. These receptors are made of five subunits arranged around a central pore. nAChRs are involved in fast neurosynaptic transmission and are thus a good molecular target for neurotoxic insecticides [17]. They are also implicated in learning and memory processes [18]. Consequently, it is not surprising that the binding of neonicotinoids to honeybee nAChRs leads to behavioral and foraging impairment [19, 20]. In honeybees, eleven genes encoding nAChR subunits have been identified, with 9 α subunits and 2 non-α (or β subunits) [21]. The subunit composition of nAChR subtypes is known to influence their pharmacological properties [20], as well as neonicotinoid binding and toxicity in honeybees [22].

In a previous study, we demonstrated that the toxic effect of neonicotinoid insecticides on the pea aphid *Acyrthosiphon pisum* is associated with differential expression of nAChR subunits after neonicotinoid exposure [23]. In this study, we further investigate the effects of a sublethal dose of SFX on *A*. *mellifera’s* learning and memory by using olfactory conditioning of the proboscis extension response (PER) paradigm. Firstly, the mortality curve from the toxicological assay allowed the sublethal dose to be determined (15 ng/bee) in order to be used in the olfactory conditioning assay. Next, we evaluated the effect of this SFX sublethal dose on learning ability, memory consolidation, and memory retrieval. In order to determine the molecular mechanisms implicated in the behavioral changes, we evaluated the changes in nAChR subunits expression levels after SFX exposure.

## Material and methods

### Honeybees

All experiments were carried out on the foraging adult honeybees *Apis mellifera*. The bees were collected between june and october 2019 from different hives located at the University of Orleans. No treatments or insecticides were applied on campus. Bees were kept in plexiglass cages (6 cm x 15 cm, 10 cm high) in groups of 30 individuals and fed with 50% (w/v) sucrose solution according to the protocol established in previous studies [24, 25]. As described in previous literature [26, 27], bees were placed in a temperature-controlled chamber at 28 ± 2°C [26].

### Determination of sulfoxaflor oral toxicity

The toxicological assay was performed according to the OECD guidelines [28] and previous studies led by Tosi and Iturbe-Requena [29, 30]. We tested a control dose (0 ng/bee) corresponding to the 50% sucrose solution, and 11 doses of SFX (15, 20, 30, 50, 100, 125, 150, 175, 200, 300 and 500 ng/bee). Bees were randomly affected to the control or to the treated groups (exposed to different SFX solutions). They were kept in a cage and were food-deprived for 2 h before the toxicological assay. Then, 10 μl of solution per bee was added to each cage using an eppendorf cap and was completely consumed within 60 min [28, 29]. After oral intoxication, honeybees were fed with 50% (w/v) sucrose solution *ad libitum* [24, 31, 32]. Mortality was recorded 48h after oral administration of SFX [29, 33]. The corrected mortality percentages were calculated according to Henderson-Tilton’s equation [34, 35] as follows:

$$\text{Corrected}\;\text{mortality}\;\%=\left(\text{1}-\frac{n\;in\;Co\;before\;treatment\times n\;in\;T\;after\;treatment}{n\;in\;Co\;after\;treatment\times n\;in\;T\;before\;treatment}\right)\times 100$$

*with n = number of honeybees*, *T = treated group*, *Co = control group*.

A mortality curve was determined using the different mortality percentages as a function of SFX dose. LD_50_ and the sublethal dose were identified as described in the statistical section.

### Olfactory conditioning of the PER

#### Honeybee sampling and selection

Adult foraging honeybees (*Apis mellifera*) were collected the evening before the test around the hives of the University of Orleans, France. Bees were anesthetized with CO_2_ and placed on ice for manipulation. Each individual was placed individually in a tube (allowing free movement of the antennae and mouthparts) and fed to satiation with a 50% (w/v) sucrose solution. Bees were maintained in an incubator at 28°C until the next day. In the morning, the responsive bees were selected for olfactory conditioning. Each bee’s antennae were stimulated with sucrose solution (50% w/v). Only bees showing PER after stimulation were kept for the following experiments. This verification stage is used to check the sensory-motor components of the PER [32, 36].

#### Olfactory conditioning

**Fig 1:**
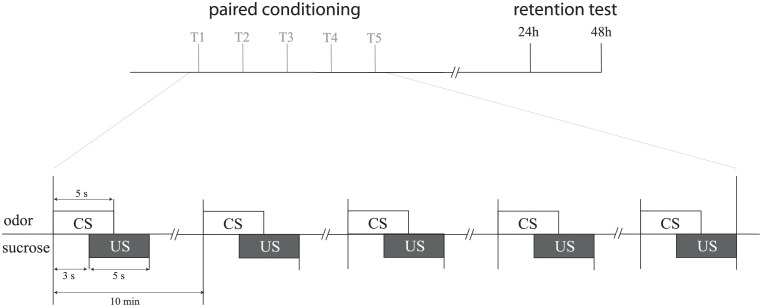
Olfactory classical PER conditioning protocol. Experimental procedure of paired group olfactory classical PER conditioning (inspired by Matsumoto et al. 2012). Lavender (conditioning stimulus, CS) was applied for 5s. After 3s, sucrose 50% (unconditioning stimulus, US) was presented. Each bee received five paired CS–US presentations (noted trial T1 to T5) with a 10 min inter-trial delay. Retention tests were performed 24 h and 48 h after the last conditioning trial and consisted of presentation of the CS without US.

Classical olfactory conditioning of the proboscis extension reflex (PER) was carried out following a well-established protocol [37, 38]. The 50% (w/v) sucrose solution was used as the unconditioned stimulus (US), with lavender being used as the conditioned stimulus (CS), as described in a recent study [39]. The CS was presented for 5s and the 50% (w/v) sucrose solution was presented for 5s, 3s after the CS [36]. Honeybees received five paired CS–US presentations with a 10 min inter-trial interval between the CS presentations [24] (Fig 1). Bees that did not extend their proboscis (PER) during conditioning were not conserved for the next steps of the experiment. According to a previous study [40], only bees that showed PER had assimilated the movement and could thus be used to quantify the process of acquisition and retrieval. The retrieval test was evaluated 24h and 48h after the end of the olfactory conditioning by presenting the CS to the bee’s antennae. Results are represented as percentage of PER in each group.

#### Honeybee exposure to sulfoxaflor before or after olfactory conditioning

Selected honeybees were randomly assigned to control and treated groups, with 30 individuals per group. The treated group was fed orally with 2 μl of 50% (w/v) sucrose solution containing 15 ng of SFX. This concentration corresponds to the NOEL (non-observed effect level) maximum dose displaying no toxicity and was defined as the sublethal dose for the next experiments [41, 42]. Note that in this experiment, the final solution of dimethyl sulfoxide (DMSO) was 0.5%. The control group was fed with 2 μl of 50% (w/v) sucrose solution containing 0.5% DMSO. To test the effect of SFX exposure on learning memory performance, honeybees were treated 30 min and 12 h before the first olfactory conditioning trial [36] or 3 h 30 and 23 h 30 after the last olfactory conditioning trial.

### Determination of nAChR subunit expression level

#### Experimental design

Quantitative PCR (qPCR) experiments were used to quantify the expression level of bee nAChR subunits, for which the nucleotide sequences have been published in a previous study [21]. Only bees showing significant alteration of the PER after SFX exposure were selected for qPCR experiments. Thus, we selected treated bees which were not able to master the olfactory conditioning, as well as treated bees which were able to learn but were unable to show a retrieval test at 24 h. Expression level in each group was determined as a relative expression ratio compared to those in the corresponding control group.

#### Total RNA extraction and cDNA preparation

Total RNA from 10 bee brains was extracted using the RNeasy micro kit (Qiagen, Courtaboeuf France) according to manufacturer recommendations. To avoid genomic DNA contamination, the protocol includes RNA treatment with DNAse I. RNA was retro-transcribed using random hexamers with ProtoScript^®^ II Reverse Transcriptase (NEB, Evry France), dissolved in RNAse-free water and conserved at -20°C.

#### Primer design and reference gene validation

Primer sets (Table 1) were designed using Primer3 software based on sequences available on the Genbank database (http://www.ncbi.nlm.nih.gov/genabnk/). Under quantitative PCR conditions, amplification efficiencies were between 80 and 110%, allowing validation of each primer set for qPCR experiments. According to the literature, 6 candidate reference genes (*rpL32*, *rpS18*, *gapdh rp49*, *RPS5* and *Tbp-af*) [43, 44] were tested for their expression stabilities with the Normfinder program [45]. *Gapdh* and *rp49* were the optimal reference genes in our conditions and were both selected for accurate normalization.

**Table 1:** Primers used to amplify nicotinic acetylcholine receptor subunits and selected reference genes in quantitative PCR experiments.

gene	Forward primer	Reverse primer	Efficiency (%)
Amelα1	CAACTACAACCGCCTCATCC	CGACACCGCCATAATCATCC	110
Amelα2	CCGACATCTTCTTCAACATCAC	AAAGCGAGCACCGATAAATAC	81
Amelα3	CGCCCTCACCGTTAAAATC	TTCCACCCCACCATATTCC	104
Amelα4	CTAACGCCAAAACGATTTCAC	CGGAGGACAGGACTTTTAAC	97
Amelα5	CGGACATCACTTACGAGATAC	AGAATACCAACAGGGCGAC	90
Amelα6	ATAGTGCCGCAAATCCTCC	ATAATCTCGTCGCTTTCATCC	92
Amelα7	AGTGATAAGGAGGAGAGGGAG	TTATTGTGGGACGCCAGAG	84
Amelα8	CGAGAAGATGATGCTCGAC	TGAGCAATAAAACGCACACC	92
Amelα9	TCTCGTCCCATCAAATCGCC	ACCCAAATATCGTCGCTCTTC	101
Amelβ1	TCCTCAAGTATCTGCCCAC	ACAACTCCATCACCTCCATC	80
Amelβ2	ATCCTCCGTCACTGAATCG	GCATAAAAAAGCACTCCATCC	83
Amel gapdh	CACCTTCTGCAAAATTATGGCG	ACCTTTGCCAAGTCTAACTGTTAA	90
Amel Rp49	CGTCATATGTTGCCAACTGGT	TTGAGCACGTTCAACAATGG	86

Gapdh = Glyceraldehyde 3-phosphate dehydrogenase; rp49 = Ribosomal protein 49

#### Quantitative PCR experiment

qPCR experiments were optimized according to MIQE Guideline recommendations [46] using the AriaMix Real-Time PCR System (Agilent, Santa Clara, USA) and GoTaq^®^ qPCR Master Mix (Promega, Fitchburg, Wisconsin USA). Experiments were performed in triplicate using 25 ng of total RNA and 150 nM of primer for a final volume of 13.5 μl. We followed standard qPCR protocol with a 10 min hot start at 95°C, 40 amplification cycles (30s at 95°C, 30s at 60°C, 1 min at 72°C), and a final melting curve determination. Product specificity was further assessed by dissociation curves giving rise to a single peak at the specific melting temperature [47]. Relative expression ratio (R) was calculated according to Pfaffl’s formula [48], using primer efficiency (E) and CP value variation between controls, and treated (ΔCP = CP control–CP treated) for each nAChR subunit. Gene expression levels after SFX exposure were calculated as a relative expression ratio, normalized using the geometric mean of the two reference genes (*gapdh* and *rp49)* and relative to control conditions, according to the following formula:

$$R=\frac{{\left(Esubunit\right)}^{\Delta CPsubunit}}{\sqrt{{\left(E\;gapdh\right)}^{\Delta CPgapdh}*{\left(E\;rp49\right)}^{\Delta CPrp49}}}$$

#### Chemicals

SFX was prepared in the CEISAM laboratory (UMR CNRS 6230, Nantes, France) as a mixture of diastereoisomers (See S1 File), following the procedure described in a previous study [49]. It was solubilized in DMSO at a final concentration of 1 mg/mL for the stock solution, as previously described [50]. For the toxicological assay, SFX was diluted in 50% (w/v) sucrose solution for all tested concentrations.

#### Statistical analysis

To estimate LD_50_ values, data were analyzed using Graphpad Prism 5 (GraphPad Software Inc., La Jolla, CA). Statistical analysis used to evaluate the effect of SFX on olfactory conditioning was described by Tison et al. [32]. During the conditioning trial and restitution test, the responses of each bee were scored as binary responses (PER, 1; no response, 0). A generalized linear mixed model analysis was applied on R software to study the relationship between PER and SFX’s effect [32, 51] (See S2 File). The best model was selected using the Akaike information criterion and validated by assessing normal Q–Q plots and residual versus fitted data plots [32, 40]. We used a χ2 test to compare control and treated groups 24 h and 48 h after olfactory conditioning. Relative expression ratios of nAChR subunit genes were compared to theoretical non-modified expression level (R = 1), (to determine whether modifications to gene expression levels were significant after SFX exposure), using a one-way ANOVA with a Dunnett post-hoc test for multiple comparison (α = 0.05) [23].

## Results

### Effect of sulfoxaflor on learning ability when administered before the conditioning

**Fig 2:**
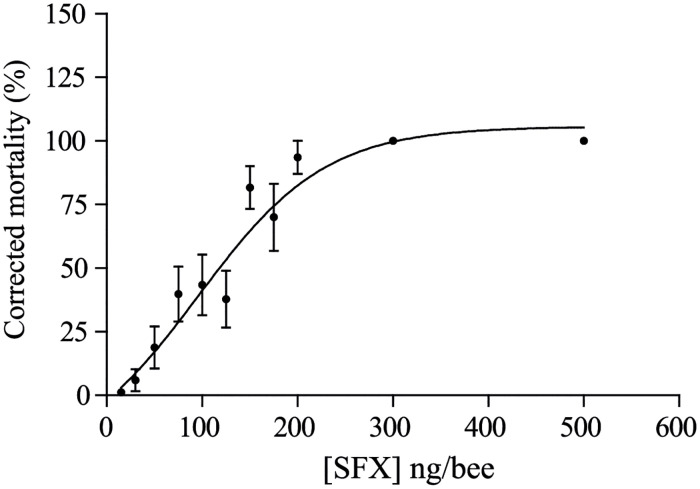
*In vivo* oral toxicity of sulfoxaflor to the honeybees, *Apis mellifera*. Toxicity curve of sulfoxaflor for adult forager honeybees. 30 bees were placed into each cage and 300 μl of the test solutions (10 μl.bee^-1^) were provided inside the cage using an eppendorf cap. SFX doses ranging from 15 ng to 500 ng/bee were tested. Mortality rate was assessed 48 h after intoxication. Each value is the mean ± SEM of three independent experiments.

The first step of our study was to determine the sublethal dose of SFX, which was used for olfactory conditioning. The toxicological assay demonstrated a dose-dependent mortality at 48 h with a LD_50_ value of 95.88 ± 0.09 ng/bee (Fig 2). Thus, as in previous studies, the NOEL was 15 ng/bee, and was defined as the sublethal dose for the next experiments [41, 42].

**Fig 3:**
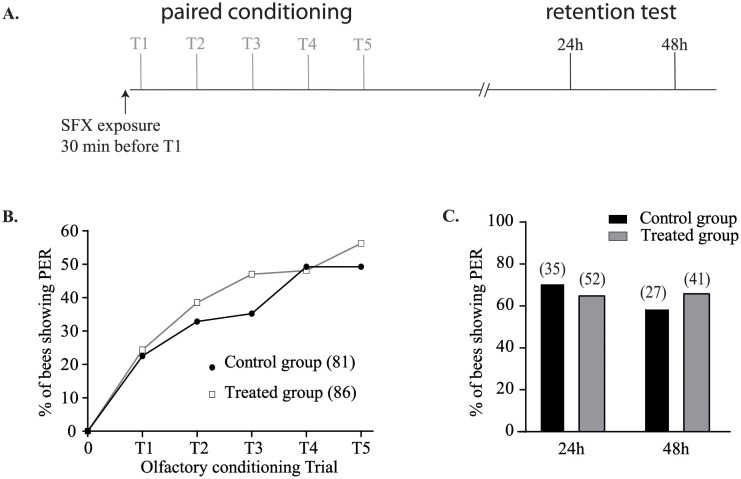
Effect of sulfoxafor sublethal dose on learning ability and memory formation after 30 minutes exposure before conditioning. **A**. Experimental procedure used: 30 min before the first olfactory conditioning trial (T1), treated and control bees received 2 μL of SFX solution (15 ng/bee) or 50% (w/v) sucrose solution respectively. **B**. The response (PER or no PER) of each bee was registered during each conditioning trial. Only surviving bees showing a proboscis extension response at the end of the olfactory conditioning were considered for the restitution test. **C**. The restitution tests were performed 24 h and 48 h after the last olfactory conditioning trial. No significant difference was observed between the treated and control groups (P > 0.05). The number of individuals in each group is given in the brackets.

Two exposure procedures were tested to assess the effect of a sublethal dose of SFX on learning and memory. In the first experiment, bees were exposed to 15 ng of SFX 30 min before the conditioning (Fig 3A). No significant difference was found during olfactory conditioning (n > 80 bees, χ^2^ = 6.35, df = 7, P = 0.09, Fig 3B), or for the retrieval performance of the PER between the control and treated groups at 24h after the conditioning (n > 35 bees, χ^2^ = 0.35, df = 1, P = 0.55, Fig 3C) and 48 h (n > 25 bees, χ^2^ = 0.3 df = 1, P = 0.58, Fig 3C).

**Fig 4:**
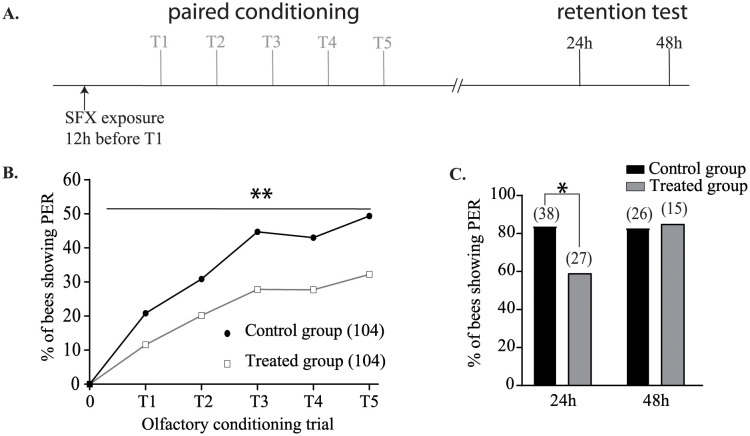
Effect of sulfoxaflor sublethal dose on learning ability and memory formation after exposure 12 h before conditioning. **A**. Experimental procedure used: 12 h before the first olfactory conditioning trial (T1), treated and control bees received 2 μL of SFX solution (15 ng/bee) or 50% (w/v) sucrose solution respectively. **B**. The response (PER or no PER) of each bee was registered during each conditioning trial. Only surviving bees showing a proboscis extension response at the end of the olfactory conditioning were considered for the restitution test. **C**. The restitution tests were performed 24h and 48h after the last olfactory conditioning trial. Asterisks indicate statistically significant differences in the treated group response compared to the control group (P < 0.05). The number of individuals in each group is given in the brackets.

To test the hypothesis of a delayed SFX effect, we investigated the impact that an acute long-term exposure of SFX had on learning and retrieval performance. Thus, bees received SFX 12h before the first conditioning trial (Fig 4A). In this condition, treated bees showed a significant decrease in their learning ability (n > 100 bees, χ^2^ = 29.98, df = 7, P < 0.001, Fig 4B). Moreover, SFX exposure led to a decrease in memory retrieval at 24h (n > 25 bees, χ^2^ = 5.09, df = 1, P = 0.024, Fig 4C). This effect was not seen at the 48h retention test (n < 30 bees, χ^2^ = 0, df = 1, P = 0.95, Fig 4C), suggesting a transient effect of SFX on the retrieval performance.

### Effect of sublethal administration of sulfoxaflor after the conditioning

**Fig 5:**
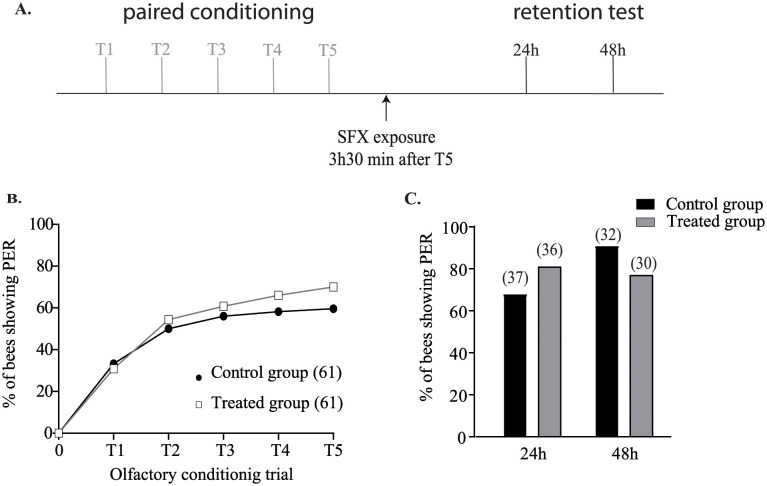
Effect of sulfoxaflor sublethal dose on memory processes after exposure 3h30 after conditioning. **A**. Experimental procedure used in protocol 3: 3 h 30 after the last olfactory conditioning trial (T5), treated and control bees received 2 μL of SFX solution (15 ng/bee) or 50% (w/v) sucrose solution respectively. **B**. The response (PER or no PER) of each bee was registered during each conditioning trial. Only survival bees showing a proboscis extension response at the end of the olfactory conditioning were considered for the restitution test. **C**. The restitution tests were performed 24 h and 48 h after the last olfactory conditioning trial. Asterisks indicate statistically significant differences in the treated group response compared to the control group (P < 0.05). The number of individuals in each group is given in the brackets.

To further investigate the impact of SFX on memory consolidation, we designed a second set of experiments. For that purpose, similarly to a previous study [40], bees were exposed to SFX 3.5 h after the last olfactory conditioning trial (Fig 5A). As expected, bees from treated and control groups had similar learning ability during the PER conditioning (Fig 5B). In addition, no significant difference between the two groups was observed during the restitution test at 24 h (n > 35 bees, χ^2^ = 1,59, df = 1, P = 0.2), or 48 h (n > 30 bees, χ^2^ = 2,23, df = 1, P = 0.13, Fig 5C).

**Fig 6:**
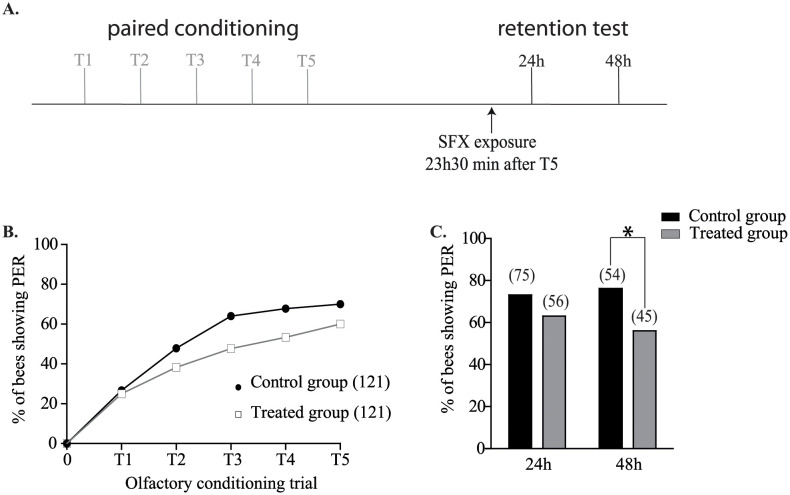
Effect of sulfoxaflor sublethal dose on memory processes after exposure 23h30 after conditioning. **A**. Experimental procedure used in protocol 4: 23h30 after the last olfactory conditioning trial (T5), treated and control bees received 2 μL of SFX solution (15 ng/bee) or 50% (w/v) sucrose solution respectively. **B**. The response (PER or no PER) of each bee was registered during each conditioning trial. Only surviving bees showing a proboscis extension response at the end of the olfactory conditioning were considered for the restitution test. **C**. The restitution tests were carried out 24 h and 48 h after the last olfactory conditioning trial. Asterisks indicate statistically significant differences in the test group response compared to the control group (P < 0.05). The number of individuals in each group is given into brackets.

These results demonstrated that bees exposed to a sublethal dose of SFX during the memory consolidation did not impair the retrieval performance. In the last set of experiments SFX was administered to bees 23.5 h after the last olfactory conditioning trial, corresponding to 30 min before the retrieval test at 24 h (Fig 6A) (Tison et al. 2017). As in previous experiments, bees from treated and control groups had similar learning ability during the olfactory PER conditioning (Fig 6B). Interestingly, during the 24 h retrieval test, no significant difference was observed between control and treated bees (n > 50 bees, χ^2^ = 1.75, df = 1, P = 0.18, Fig 6C), but a significant difference was found at 48 h after the conditioning (n > 40 bees, χ^2^ = 4.58, df = 1, P = 0.03, Fig 6C). These results sustained the hypothesis that SFX has a delayed effect on bee memory retrieval.

### Sulfoxaflor effect on nicotinic acetylcholine receptor subunit expression in the honeybee brain

**Fig 7:**
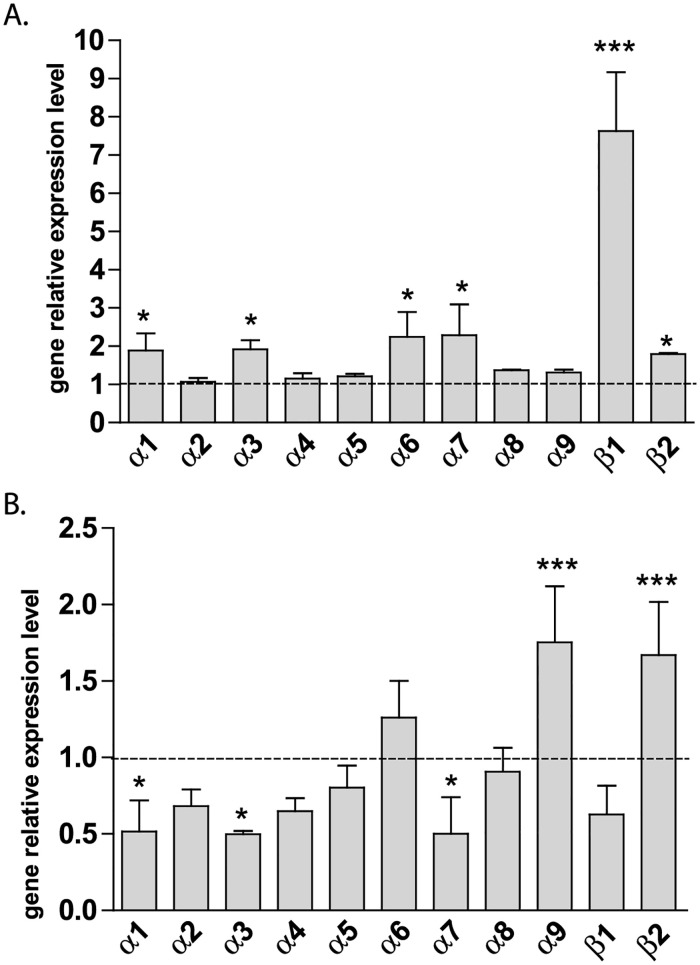
Expression levels of nAChR mRNA subunits in honeybee brains. **A**. relative expression level of nAChR subunits in bees with learning ability impairment after sulfoxaflor exposure. Experiments were based on honeybees exposed to sulfoxaflor 12h before conditioning that were unable to learn at the end of olfactory conditioning. **B**. relative expression level of nAChR subunits in bees with memory retrieval impairment after sulfoxaflor exposure. Experiments were based on honeybee exposed to sulfoxaflor 23 h 30 after conditioning (i.e. 30 min before the 24 h retrieval test) that did not show PER during the retention test. For all experiments, results are expressed as a percentage of the control. For each gene, the expression levels in the control group corresponds to 100% and is represented by a dotted line. Each qPCR experiment was performed in triplicate and results are the mean of four independent experiments. Relative expression ratios were normalized using two reference genes, *gapdh* and *rp49*. Error bars represent the SEM. Statistical analysis (t-test, α = 0.05) was carried out using Graphpad Prism 5 software and results statistically different from control are marked with an asterisk.

In order to investigate the link between SFX impairment on learning and memory, and bee nAChR expression, we quantified the relative expression level of genes encoding nAChR subunits. We therefore selected treated bees that were not able to learn during olfactory conditioning (Fig 4) in order to identify possible modifications to gene expression levels compared to the control bees that learned correctly. In this condition, we observed high variations in gene expression levels in treated bees compared to the control group (Fig 7A). In particular, Amelβ1 expression was 762 ± 30% higher in the treated group compared to the control. We also observed a significant increase in Amelα6 (+123 ± 12%), Amelα7 (+129 ± 16%), Amelα3 (+91 ± 4%), Amelα1 (+89 ± 8%) and Amelβ2 (+79 ± 5%). These results suggested that modifications to the subunit expression could be linked to the learning defect induced by SFX. Considering this hypothesis, the same approach was applied to treated bees that did not demonstrate PER during the 48 h retrieval test (see Fig 6). Compared to control bees that demonstrated correct memory retrieval, bees from this experiment presented significant modifications to gene expression levels (Fig 7B). Indeed, we found that three nAChR subunit genes were down-regulated during the retrieval performance. This was the case for Amelα1 (-49 ± 2%), Amelα3 (-51 ± 0.2%) and Amelα7 (-49 ± 2%), whereas Amelα9 (+75 ± 4%) and Amelβ2 (+67 ± 3%) were up-regulated in bees exposed to SFX, confirming that SFX application to bees alters behavioral performance and modifies bee nAChR subunit expression.

## Discussion

### Effects of a sublethal dose of SFX on learning and memory performance

Firstly, we evaluated the *in vivo* toxicity of SFX to the honeybee *Apis mellifera*. The mortality curve enabled us to estimate the LD_50_ of SFX as being 96 ng/bee. This result is slightly lower than that presented in the EFSA report, which indicated an LD_50_ of 146 ng/bee after acute oral exposure [52], which can be linked to the fact that in our experiments, we only used summer forager bees, which may have different genotypes or ages. Indeed, a previous study highlighted high variations in neonicotinoid toxicity, with IMI having an LD_50_ between 2.5 ng/bee and 83.3 ng/bee, depending on honeybee genotype and age [53]. In addition, we also found that SFX was less toxic to bees than neonicotinoid insecticides such as IMI, CLT or TMX [54]. This toxicological assay enabled us to determine the sublethal dose used in learning and memory tests which was selected as the sublethal dose for the PER experiments. Our results indicated that a sublethal dose of SFX can affect learning and memory processes in different ways depending on the exposure protocol. In the first set of experiments, we found a strong SFX effect when bees were intoxicated 12h before the olfactory conditioning, but no effect was observed when SFX exposure took place 30 min before conditioning. In a previous study, Siviter et al. (2019) demonstrated that SFX did not impair learning performance when honeybees were exposed to very low doses just before olfactory training [39]. The discrepancy in our study could be associated to the finding that we used a higher concentration of SFX compared to the study by Siviter et al. [39]. Again, such variability could be linked to different genotype backgrounds in bees [53]. Interestingly, a similar learning defect was observed when bees were exposed to high concentrations of the neonicotinoid thiacloprid (69 ng/bee) just before olfactory conditioning [40] and IMI (12 ng/bee) [36], demonstrating that the insecticide dose being tested is a critical parameter. Another hypothesis is that the delay between intoxication and conditioning trials is crucial for observing the SFX effect on learning ability. Indeed, we also noticed a decrease in the restitution rate at 24 h when SFX was administrated 12 h before the conditioning, demonstrating that early exposure to an SFX sublethal dose could also impair memory retrieval. We observed a spontaneous recovery in the memory retrieval of treated bees at 48 h, demonstrating that memory formation was not impaired but was not accessible after SFX exposure. Similar results have been demonstrated previously after a sublethal exposure of CLT [32] and IMI [55].

Moreover, we propose that SFX exposure during memory consolidation did not influence memory retrieval performances. On the contrary, SFX intoxication just before the restitution test led to memory retrieval impairment at 48h. These results suggest that i) some stages of learning and memory processes are more sensitive to SFX than others, and ii) in terms of learning ability, the effect of SFX on memory retrieval probably involves molecular mechanisms, which justifies the delay between SFX exposure and retrieval defect. These results are consistent with previous studies, demonstrating a memory impairment due to neonicotinoid exposure [32, 36, 40, 56]. Indeed, CLT at 0.3 ng/bee and 0.8 ng/bee induced a decrease in memory retention and interfered with memory retrieval [32]. A recent study demonstrated that IMI at a sublethal dose (0.12 ng/bee) induced a negative effect on medium-term retention, but not on the short and long-term retention. The authors proposed that IMI could act on memory formation or memory restitution [36]. In the same way, Tison et al. (2017) showed a significant reduction in retention during the memory tests at 24 h after THC intake at high doses (20, 69 and 200 ng/ bee) [40]. Altogether, these results demonstrate the impact of a sublethal dose of SFX on bee behavior. Similar effects were reported after intoxication with neonicotinoids, such as TMX, which induced either a significant decrease in olfactory learning ability or memory retrieval performance depending on exposure protocol [57].

### Involvement of honeybee nAChRs in the SFX effect

nAChRs are involved in the various phases of classical olfactory conditioning [18, 58, 59], and previous studies highlighted the link between modulation of nAChR activity and impairment of learning and memory [60, 61]. We proposed that the differential effect of SFX observed depending on the exposure procedure could be due to its action on specific nAChRs that are differently implicated in memory formation and retrieval processes. In fact, previous studies demonstrated that α-bungarotoxin-sensitive nAChRs are involved in memory consolidation, whereas memory retrieval is affected by mecamylamine-sensitive nicotinic receptors [60–63]. To further assess the mechanism underlying the effect of SFX on learning and memory processes, we determined the variation in nAChR subunit expression for honeybee brains presenting a degradation in either their learning or retrieval ability after SFX exposure. In these two groups of intoxicated bees, we observed significant modulations to nAChR subunit expression levels. This is in line with previous studies demonstrating neuronal plasticity, and nAChR expression modifications associated with chronic oral exposure to neonicotinoids in adult honeybees [19]. In our study, three α subunits (Amelα1, Amelα3, Amelα7) seem to be regulated in the same way after SFX exposure procedures. These α subunits are up-regulated in SFX-treated bees with learning defects, and down-regulated in SFX-treated bees with retrieval impairment. The modulation to Amelα1 subunit expression is consistent with a recent publication showing that Amelα1 was upregulated after exposure to CLT, IMI, or TMX after chronic oral exposure to neonicotinoids at low doses [50]. In their study, Christen et al. (2016) identified an increase in Amelα1 and Amelα2 subunit expression after 48 h, and no modification at 72 h [50]. We also observed an overexpression of the Amelα9 and Amelβ2 subunits in intoxicated bees with retrieval defects. This is consistent with a previous study which highlighted a similar increase in these subunits after 10 days of exposure to TMX [64]. Changes in nAChR subunit expression after exposure to neonicotinoids have also been described in other insects such as the pea aphid *Acyrthosiphon pisum* [23], the planthopper *Nilaparvata lugens* [65], the house fly *Musca domestica* [66], and the cockroach *Periplaneta americana* [67]. This regulation of nAChR subunit expression is considered as a compensatory mechanism for the decrease in ACh-sensitivity of the receptors [19].

Gene expression patterns seem to present specific modifications depending on the insecticide tested. For example, exposure to IMI and TMX also induced the expression of Amelα2 [50], which is not the case in our experiments with SFX. We also observed overexpression of Amelβ1 and Amelβ2 after SFX intoxication, whereas IMI exposure is associated with a decreased expression of these subunits in honeybees [68]. This is in accordance with recent studies on cockroach DUM neurons, suggesting that SFX acts on nAChR subtypes distinct from those implicated in the interaction of neonicotinoids such as IMI or TMX [69–71]. At the molecular level, a recent study on *drosophila* suggested that SFX could bind to nAChR subtypes including the Dmelβ1 subunit [20]. We propose that Amelβ1 expression could also make honeybees have an increased sensitivity to SFX. Moreover, Amelα7 was respectively up-regulated or down-regulated in SFX-intoxicated bees presenting olfactory learning or memory retrieval defects. These data suggest that receptors containing Amelα7 have a different role in learning and memory processes. Besides, Amelα1 and Amelα3 are co-regulated, suggesting that these two subunits belong to the same receptor subtypes. Their low expression levels are correlated with retrieval defects induced by SFX, and these subunits were previously co-localized with Amelβ1 in kenyon cells and antennal lobes [72–74]. Thus, we propose that heteromeric nAChRs consisting of Amelα1 Amelα3 and Amelβ1 or homomeric Amelα7 nAChRs could be implicated in the memory retrieval process in honeybees. In fact, α7 subunits are able to form functional homomeric receptors, as demonstrated in *D*. *melanogaster* [75]) and *P*. *americana* [76]. A previous study also demonstrated that Amelα8 subunits from the mushroom bodies are implicated in memory retrieval [77]. Therefore, various nAChR subtypes are probably involved in both learning and memory processes, and SFX’s mode of action. The molecular composition of nAChRs must still be elucidated, and further investigations are needed in order to understand the molecular mechanisms underlying the effect of an SFX sublethal dose on learning and memory in bees.

## Conclusion

In this study, by using the PER paradigm, we demonstrated previously undiscovered learning and memory impairment after exposure to a sublethal dose of sulfoxaflor. In particular, we demonstrated that the SFX effect is displayed with a delay between exposure and behavioral impairment. To explore SFX’s mode of action, we identified the modulations to the expression pattern of the nAChR subunits following SFX exposure. We found that sulfoxaflor exposure led to over- or under-expression of several nAChR subunits, which are specific depending on the exposure protocol. As nAChRs play an important role in learning and memory, their modulation could be (at least in part) responsible for the defects in learning ability and memory formation that were observed. In all cases, we demonstrated that SFX is able to impair honeybee learning and memory performance. Further characterization of nAChR subtypes involved in the response to SFX exposure are needed in order to better understand sulfoxaflor’s impact on honeybee behavior. As sulfoxaflor is a candidate for replacing neonicotinoid insecticides in crop protection strategies, our results highlight the need to better understand sulfoxaflor’s mode of action in order to correctly assess the environmental risk to pollinators.

## Supporting information

S1 File Synthesis of sulfoxaflor. (DOCX)

S2 File Script for statistic test. (DOCX)

S1 Graphical abstract (EPS)
