# Supplementary material for: Impairments in learning and memory performances associated with nicotinic receptor expression in the honeybee Apis mellifera after exposure to a sublethal dose of sulfoxaflor
Source: PLoS One. 2022 Aug 3;17(8):e0272514. doi: 10.1371/journal.pone.0272514 (PMC9348702; doi:10.1371/journal.pone.0272514)
Supplement: S1 File — (DOCX) [file pone.0272514.s001.docx]

**Impairments in learning and memory performances associated with nicotinic receptor expression in the honeybee *Apis mellifera* after exposure to a sublethal dose of sulfoxaflor.**

Alison Cartereau^1^, Xavier Pineau^1^, Jacques Lebreton^2^, Monique Mathé-Allainmat^2^, Emiliane Taillebois^1^, Steeve H. Thany^1*^

^1^Université d’Orléans, Laboratoire de Biologie des Ligneux et des Grandes Cultures (LBLGC) USC INRAE 1328, 1 rue de Chartres, 45067 Orléans, France.

^2^ Nantes Université, CEISAM UMR CNRS 6230, UFR des Sciences et des Techniques, 2 rue de la Houssinière, BP 92208, 44322 Nantes, France.

**Table of contents :**

- Chemistry general and SFX synthetic scheme S2
- Synthesis of 5-(1-(Methylthio)ethyl)-2-(trifluoromethyl)pyridine **2** S3
- Synthesis of 5-(1-(Methylsulfinyl)ethyl)-2-(trifluoromethyl)pyridine **3** S3
- Synthesis of 2,2,2-Triﬂuoro-N-(S-methyl-(1-(2-triﬂuoromethylpyridin-5-yl)ethyl)
- oxido-λ6-sulfanylidene)acetamide **4** S4
- Synthesis of 5-(1-(S-methylsulfonimidoyl)ethyl)-2-(trifluoromethyl)pyridine **5** S4
- *Synthesis of [methyl(oxo){1-[6-(trifluoromethyl)-3-pyridyl]ethyl}-λ6-sulfanylidene]*

*cyanamide*, SULFOXAFLOR, **6** S5

- ^1^H NMR ^13^C NMR and ^19^F NMR spectra of compound **3** S6
- ^1^H NMR ^13^C NMR and ^19^F NMR spectra of compound **4** S8
- ^1^H NMR ^13^C NMR and ^19^F NMR spectra of compound **5** S10
- ^1^H NMR ^13^C NMR and ^19^F NMR spectra of SULFOXAFLOR **6** S12

*General:* All solvents used were reagent grade. TLC was performed on silica-covered aluminum sheets (Kieselgel 60F_254_, MERCK). Eluted TLC was revealed using UV or molybdate solution. Flash column chromatography was performed on silica gel 60 ACC 40-63 µm (SDS-CarloErba). NMR spectra were recorded on a BRUKER 300 or a BRUKER 400 at room temperature, on samples dissolved in an appropriate deuterated solvent. References of tetramethylsilane (TMS) for ^1^H and deuterated solvent signal for ^13^C were used. Chemical displacement values (*δ*) are expressed in parts per million (ppm), and coupling constants (*J*) in Hertz (Hz). High-Resolution Mass Spectrometry (HRMS in Da unit) analyses were done on a Waters Xevo G2-XS QTOF on the AMaCC platform in CEISAM laboratory. Hight Performance Liquid Chromatography (HPLC) analyses were done on a chiral HPLC 1200 Agilent on the AMaCC platform in CEISAM laboratory.

*Scheme 1* : Synthesis of SULFOXAFLOR following procedures described in the literature.^[[1]](#footnote-1)^ ^[[2]](#footnote-2)^

*Synthesis of 5-(1-(Methylthio)ethyl)-2-(trifluoromethyl)pyridine* ***2***

|  | **Formula :** C_9_H_10_F_3_NS  **MW :** 221.24 g.mol^-1^ |
| --- | --- |

A solution of (E)-1-(3-(methylthio)but-1-en-1-yl)pyrrolidine **1** (4.39 g, 25.63 mmol, 1 eq) and (E)-4-ethoxy-1,1,1-trifluorobut-3-en-2-one (4.74 g, 4.02 mL, 28.20 mmol, 1.1 eq) in acetonitrile (10 mL) was stirred overnight under argon atmosphere at room temperature. Ammonium acetate (2.96 g, 38.45 mmol, 1.5 eq) was then added and the mixture was stirred for an additional hour at 80°C. Cyclohexane (200mL) and water
(200 mL) were added. The organic layer was washed with brine, dried over MgSO_4_, filtrated and evaporated under vacuum. The crude product was purified by column chromatography on silicagel (petroleum ether/EtOAc ; 9 :1) to afford the product **2** as a yellow liquid (67 %).

**^1^H NMR (300 MHz, CDCl_3_) :** δ 8.65 (d, 1H, 2.1 Hz, H_ar_) ; 7.88 (dd, 1H, 8.1 Hz and 2.1 Hz, H_ar_) ; 7.66 (d, 1H, 8.1 Hz, H_ar_) ; 3.92 (q, 1H, 7.2 Hz, CH) ; 1.93 (s, 3H, SCH) ; 1.61 (d, 3H, 7.2 Hz, CH_3_).

**^19^F NMR (376 MHz, CDCl_3_) :** δ - 67.81.

**^13^C NMR (75 MHz, CDCl_3_) :** δ 149.3 (CH_ar_) ; 147.0 (q, *J* = 33.75 Hz, Civ_ar_) ; 142.8 (Civ_ar_) ; 135.9 (CH_ar_) ; 121.5 (q, *J* = 272.25 Hz, CF_3_) ; 120.4 (q, *J* =2.25 Hz, CH_ar 3_) ; 42.7 (CH) ; 21.7 (CH _3_) ; 14.5 (SCH _3_).

**MS**  (ESI+) : [M+H]^+^ = 222.1.

**HRMS** (ESI+) calculated for C_9_H_11_F_3_NS [M+H]^+^ m/z = 222.0559; found 222.0556.

*Synthesis of 5-(1-(Methylsulfinyl)ethyl)-2-(trifluoromethyl)pyridine* ***3***

|  | **Formula :** C_9_H_10_F_3_NOS  **MW :** 237.24 g.mol^-1^ |
| --- | --- |

To a solution of **2** (3.2 g, 14.46 mmol, 1 eq) in methylene chloride (50 mL) was added *m*-chloroperbenzoic acid (mCPBA)(2.5 g, 14.46 mmol, 1 eq). The mixture was stirred overnight under argon atmosphere at room temperature and hydrolyzed with an aqueous solution of sodium hydrogenocarbonate. The organic layer was washed with water, brine, dried over MgSO_4_, filtrated and evaporated under vacuum. The crude product was purified by column chromatography on silicagel (DCM/MeOH ; 10 :0 to 95 :5) to afford the product **3** as a yellow liquid (50 %).

**^1^H NMR (400 MHz, CDCl_3_) :** *first diastereoisomer* - δ 8.64 (d, 1H, *J*  = 2.0 Hz, H_ar_) ; 7.82 (dd, 1H, *J* = 8.0 Hz and 2.0 Hz, H_ar_) ; 7.71 (d, 1H, *J* = 8.0 Hz, H_ar_) ; 3.93 (q, 1H, *J* = 7.2 Hz, CH) ; 2.35 (s, 3H, SOCH_3_) ; 1.72 (d, 3H, *J* = 7.2 Hz, CH_3_). *Second diastereoisomer* - δ 8.59 (d, 1H, *J*  = 2.0 Hz, H_ar_) ; 7.89 (dd, 1H, , *J* = 8.0 Hz and 2.0 Hz, H_ar_) ; 7.73 (d, 1H, *J* = 8.0 Hz, H_ar_) ; 3.77 (q, 1H, *J* = 7.2 Hz, CH) ; 2.25 (s, 3H, SOCH_3_) ; 1.77 (d, 3H, 7, *J* = 7.2 Hz, CH_3_).

**^19^F NMR (376 MHz, CDCl_3_) :** δ -67.96 ; -67.99.

**^13^C NMR (101 MHz, CDCl_3_) :** *First diastereoisomer* - δ 149.6 (CH_ar_) ; 148.3 (q, *J* = 35.1 Hz, Civ_ar_) ; 137.1 (CH_ar_) ; 135.1 (Civ_ar_) ; 121.3 (q, *J* = 272.5 Hz, CF_3_) ; 120.5 (q, *J* = 2.5 Hz, CH_ar_ ) ; 59.9 (CH) ; 35.3 (SOCH_3_) ; 13.4 (CH_3_). *Second diastereoisomer* - δ 150.1 (CH_ar_) ; 148.2 (q, *J* = 35.1 Hz, Civ_ar_) ; 137.5 (CH_ar_) ; 133.8 (CH_ar_) ;121.4 (q, *J* = 272.5 Hz, CF_3_) ; 120.4 (q, *J* = 2.5 Hz, CH_ar_) ; 57.7 (CH) ; 35.9 (SOCH_3_) ; 15.1 (CH_3_).

**HRMS** (ESI+) calculated for C_9_H_10_F_3_NNaOS [M+Na]^+^ m/z = 260.0327; found 260.0323.

*Synthesis of 2,2,2-trifluoro-N-(methyl(oxo)(1-(6-(trifluoromethyl)pyridin-3-yl)ethyl)-λ^6^-sulfaneylidene)acetamide* ***4***

|  | **Formula :** C_11_H_10_F_6_N_2_O_2_S  **MW :** 348,27 g.mol^-1^ |
| --- | --- |

To a solution of 5-(1-(methylsulfinyl)ethyl)-2-(trifluoromethyl)pyridine **3** (1.52 g, 6.43 mmol, 1 eq) in methylene chloride (50 mL) were added trifluoroacetamide (1.45 g, 12.86 mmol, 2 eq), magnesium oxide (1.04 g, 25.71 mmol, 4 eq), rhodium(II) acetate dimer (142 mg, 0.32 mmol, 5mol%) and diacetoxy iodo-benzene (PIDA) (3.31 g, 10.28 mmol, 1.6 eq). The mixture was stirred overnight under argon atmosphere at room temperature, filtrated over celite and evaporated under vacuum. The crude product was purified by column chromatography (DCM/MeOH ; 10 :0 to 9 :/5) to afford the product **4** as a yellow liquid as a mixture of diastereoisomers in a ratio of 2 :1 (89 %).

**^1^H NMR (400 MHz, CDCl_3_) :** *Diastereoisomer* (mino)- δ 8.80 (d, 1H, *J* = 2.0 Hz, CH_ar_) ; 8.09 (dd, 1H, *J* = 8.0 Hz and 2.0 Hz, CH_ar_) ; 7.80 (d, 1H, *J* = 8.0 Hz, CH_ar_) ; 4.89 (q, 1H, *J* = 7.2 Hz, CH) ; 3.30 (s, 3H, SOCH_3_) ; 1.92 (d, 3H, *J* = 7.2 Hz, CH_3_). *Diastereoisomer* (majo)- δ 8.77 (d, 1H, *J* = 2.0 Hz, CH_ar_) ; 8.06 (dd, 1H, *J* = 8.0 Hz and 2.0 Hz, CH_ar_) ; 7.77 (d, 1H, *J* = 8.0 Hz, CH_ar_) ; 4.87 (q, 1H, *J* = 7.2 Hz, CH) ; 3.30 (s, 3H, SOCH_3_) ; 1.97 (d, 3H, *J* = 7.2 Hz, CH_3_).

**RMN ^19^F (376 MHz, CDCl_3_) :** δ -68.16 (CF_3_ both dias); -75.88 (COCF_3_ dia mino); -75.99 (COCF_3_ dia majo).

**RMN ^13^C (101 MHz, CDCl_3_) :** *Diastereoisomer majo* - δ 164.0 (q, *J* = 39 Hz, *C*OCF_3_) ; 150.8 (CH_ar_) ; 149.9 (q, *J* = 35 Hz, Civ_ar_) ; 138.7 (CH_ar_) ; 131.0 (Civ_ar_) ; 121.1 (q, *J* = 272.2 Hz, CF_3_) ; 120.9 (q, *J* = 2.4 Hz, CH_ar_) ; 115.7 (q, *J* = 285.4 Hz, CF_3_) ; 61.6 (CH) ; 36.6 (SOCH_3_) ; 13.6 (CH_3_). *Diastereoisomer* (mino)- δ 164.0 (q, *J* = 39 Hz, CO) ; 150.6 (CH_ar_) ; 149.7 (q, *J* = 35 Hz, Civ_ar_) ; 138.7 (CH_ar_) ; 130.7 (Civ_ar_) ; 121.0 (q, *J* = 272.2 Hz, CF_3_) ; 120.7 (q, *J* = 2.4 Hz, CH_ar_) ; 115.5 (q, *J* = 285.4 Hz, CF_3_) ; 62.0 (CH_'_) ; 36.5 (SOCH_3_) ; 13.7 (CH_3_).

HRMS (ESI+) calculated for C_11_H_11_F_6_N_2_O_2_S [M+H]^+^ m/z = 349.0440; found 349.0442.

*Synthesis of 5-(1-(S-methylsulfonimidoyl)ethyl)-2-(trifluoromethyl)pyridine* ***5***

|  | **Formule :** C_9_H_11_F_3_N_2_OS  **Masse molaire :** 252.26 g.mol^-1^ |
| --- | --- |

To a solution of **4** (1 g, 2.87 mmol, 1 eq) in methanol (50 mL) was added potassium carbonate (317 mg, 2.30 mmol, 0.8 eq). The mixture was stirred during 7 h at room temperature, concentrated under reduced pressure and water was added. The product was extracted with ethyl acetate and the organic layer was washed with water, brine, dried over MgSO_4_, filtrated and evaporated under vacuum to give compound **5** as a yellow liquid (diastereoisomer ratio 1 :1) used in the next step without purification (quantitative, residue of solvents).

**^1^H NMR (400 MHz, CDCl_3_) :** *First diastereoisomer* - δ 8.75 (d, 1H, *J* = 2.0 Hz, CH_ar_) ; 8.05 (dd, 1H, *J* = 8.0 Hz and 2.0 Hz, CH_ar_) ; 7.74 (d, 1H, *J* = 8.0 Hz, CH_ar_) ; 4.37 (q, 1H, *J* = 7.2 Hz, CH) ; 2.86 (s, 3H, SOCH_3_) ; 1.83 (d, 3H, *J* = 7.2 Hz, CH_3_). *Second diastereoisomer* - δ 8.74 (d, 1H, *J* = 2.0 Hz, CH_ar_) ; 8.02 (dd, 1H, *J* = 8.0 Hz and 2.0 Hz, CH_ar_) ; 7.74 (d, 1H, *J* = 8.0 Hz, CH_ar_) ; 4.32 (q, 1H, *J* = 7.2 Hz, CH) ; 2.90 (s, 3H,SOCH_3_) ; 1.85 (d, 3H, *J* = 7.2 Hz, CH_3_).

**^19^F NMR (376 MHz, CDCl_3_) :** -68.02, -68.02.

**^13^C NMR (101 MHz, CDCl_3_) :** *First diastereoisomer* - δ 150.6 (CH_ar_) ; 148.6 (m, Civ_ar_) ; 137.9 (CH_ar_) ; 133.6 (Civ_ar_) ; 121.3 (q, *J* = 271.9 Hz, CF_3_) ; 120.5 (CH_ar_) ; 63.9 (CH) ; 40.0 (SOCH_3_) ; 14.25 (CH_3_). *Second diastereoisomer* - δ 150.5 (CH_ar_) ; 149.4 (m, Civ_ar_) ; 138.0 (CH_ar_) ; 133.8 (Civ_ar_) ; 121.3 (q, *J* = 271.9 Hz, CF_3_) ; 120.5 (CH_ar_) ; 64.0 (CH) ; 40.2 (SOCH_3_) ; 15.0 (CH_3_).

*Synthesis of [methyl(oxo){1-[6-(trifluoromethyl)-3-pyridyl]ethyl}-λ6-sulfanylidene]cyanamide, SULFOXAFLOR,* ***6***

|  | **Formula :** C_10_H_10_F_3_N_3_OS  **MW :** 277.27 g.mol^-1^ |
| --- | --- |

To a solution of **5** (0.1 g, 0.4 mmol, 1 eq) in DCM (5 mL) was added under argon DMAP (5 mg, 0.04 mmol, 0.1 eq), Et_3_N (0.16 mL, 0.72 mmol, 1.8 eq) and then BrCN (42 mg, 0.4 mmol, 1 eq) at 0 °C . The mixture was stirred during 16 h at room temperature, then diluted with DCM and extracted with aqueous solution of HCl (2N), saturated solution of NaHCO_3_, dried over MgSO_4_, filtrated and evaporated under vacuum. The crude was purified by column chromatography on silicagel (eluent DCM/MeOH, 95 :5), to give the expected product **6** as a mixture of diastereoisomer (ratio 1 : 1) and as a white powder (27 %).

**^1^H NMR (400 MHz, CDCl_3_) :** *First diastereoisomer* - δ 8.79 (d, 1H, *J* = 2.0 Hz, CH_ar_) ; 8.05 (m, 1H, CH_ar_) ; 7.82 (d, 1H, *J* = 8.0 Hz, CH_ar_) ; 4.68 (q, 1H, *J* = 8 Hz, CH) ; 3.11 (s, 3H, SOCH_3_) ; 2.01 (d, 3H, CH_3,_). *Second diastereoisomer* - δ 8.78 (d, 1H, *J* = 2.0 Hz, CH_ar_) ; 8.05 (m, 1H, CH_ar_) ; 7.82 (d, 1H, *J* = 8.0 Hz, CH_ar_); 4.68 (q, 1H, *J* = 8 Hz, CH) ; 3.07 (s, 3H, SOCH_3_) ; 2.01 (d, 3H, CH_3,_)

**RMN ^19^F (376 MHz, CDCl_3_) :** -68.16 ; -68.17

**RMN ^13^C (101 MHz, CDCl_3_)** *some* *quaternary carbons are missing***:** *First diastereoisomer* - δ 150.50 (CH_ar_) ; 138.52 (CH_ar_) ; 121.3 (q, 271.9 Hz, CF_3_) ; 130.7 ; 121.14-121.0 (CH_ar_ and CN) ; 111.14 (CN) ; 64.02 (CH) ; 38.27 (SOCH_3_) ; 14.05 (CH_3_). *Second diastereoisomer* - δ 150.45 (CH_ar_) ; 138.45 (CH_a_) ; 130.7 (CH_ar_) ; 121.14-121.0 (CH_ar_ and CN); 111.14 (CN) ; 63.95 (CH) ; 37.78 (SOCH_3_) ; 13.96 (CH_3_).

**HRMS** (ESI-) calculated for C_10_H_9_F_3_N_3_OS [M-H]^-^ *m/z* = 276.0418; found 276.0421. **HRMS (ESI+)** calculated for C_10_H_10_F_3_N_3_ONaS [M+Na]^+^ *m/z* = 300.0394; found 300.0405.

^1^H NMR, ^13^C NMR and ^19^F NMR spectra of compound **3** (400 MHz, CDCl_3_)

|  |  |
| --- | --- |


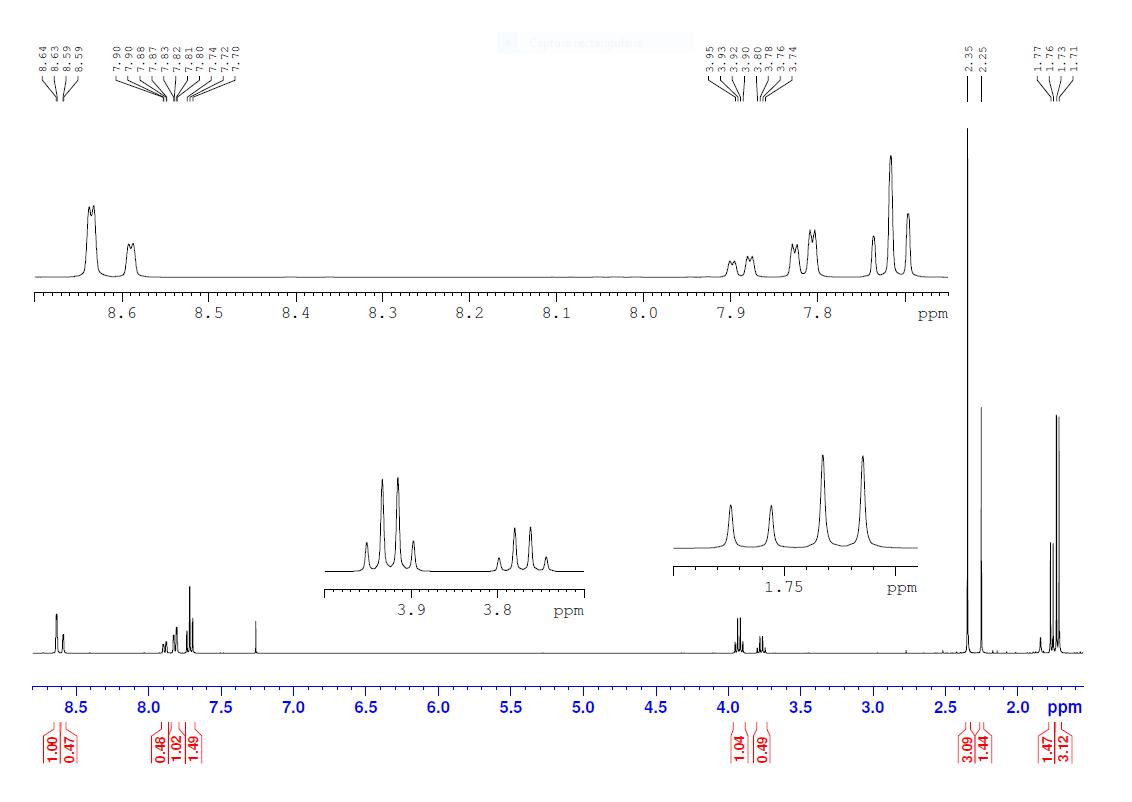


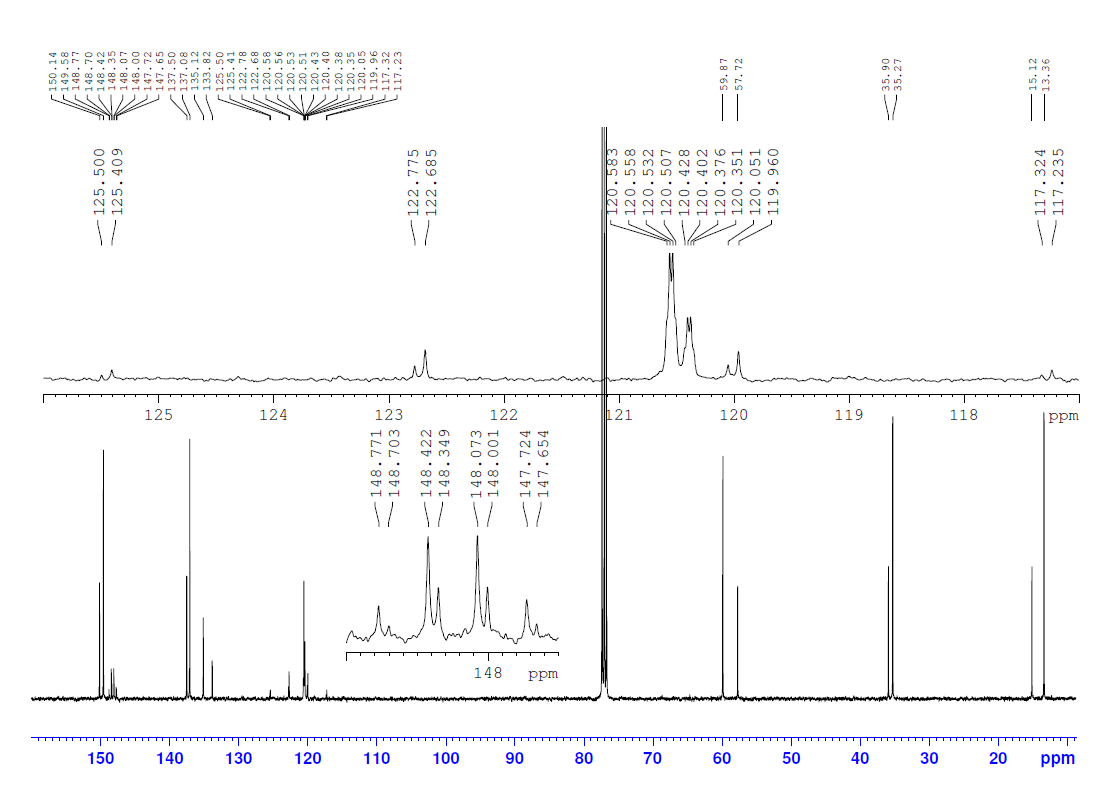


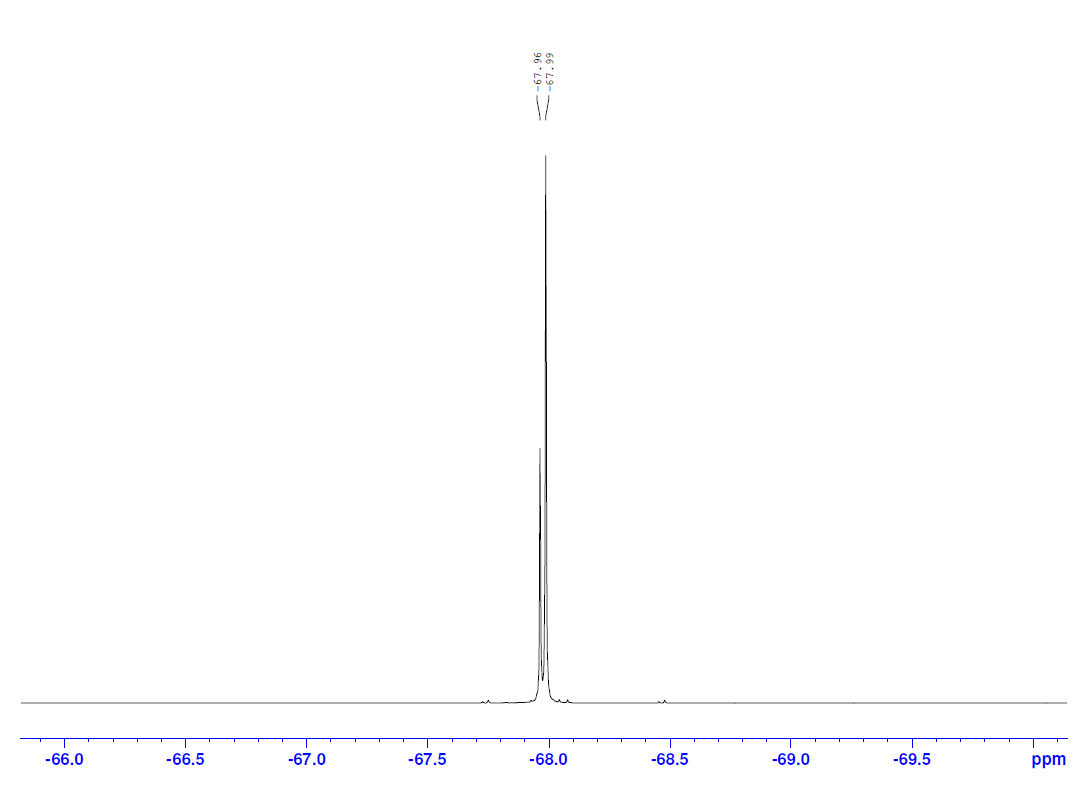


^1^H NMR, ^13^C NMR and ^19^F NMR spectra of compound **4** (400 MHz, CDCl_3_)

|  |  |
| --- | --- |


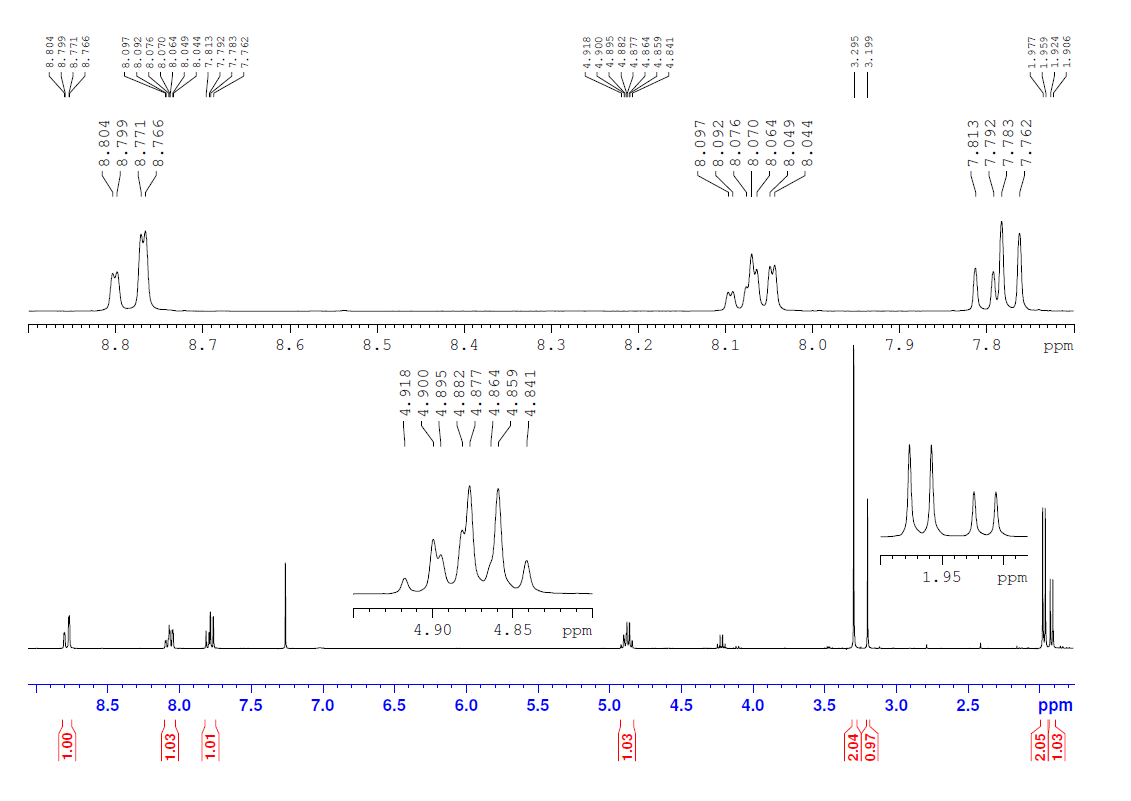


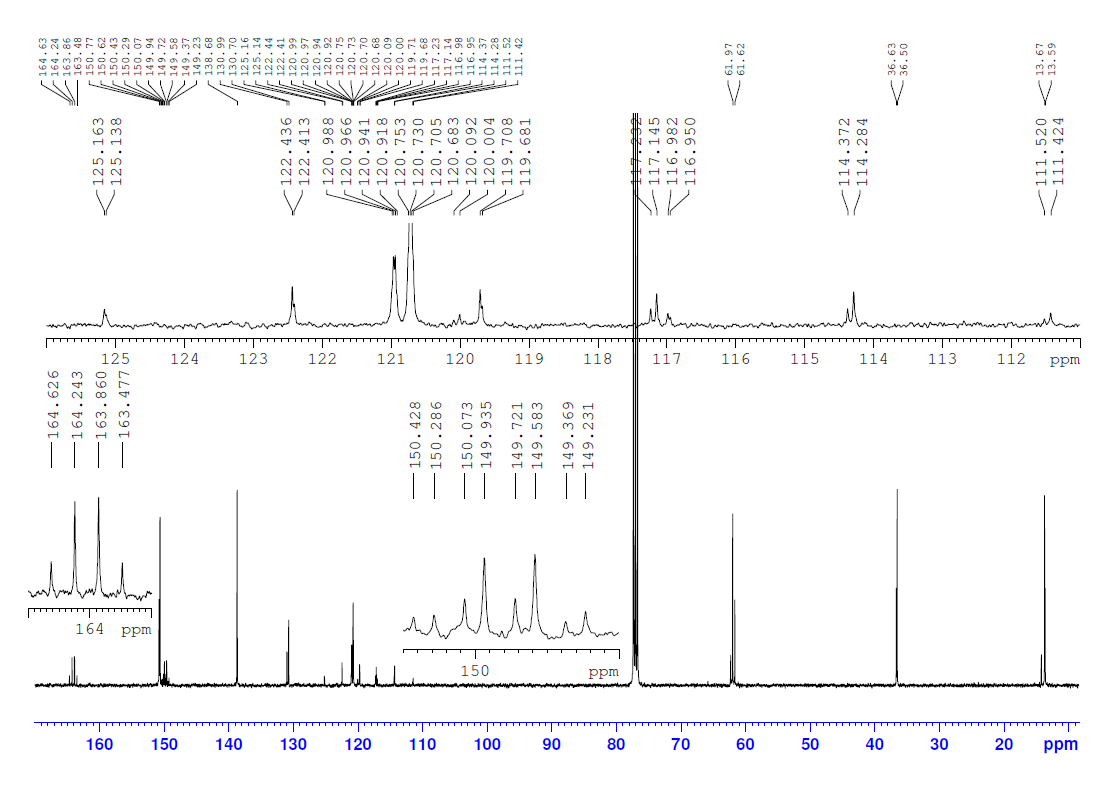


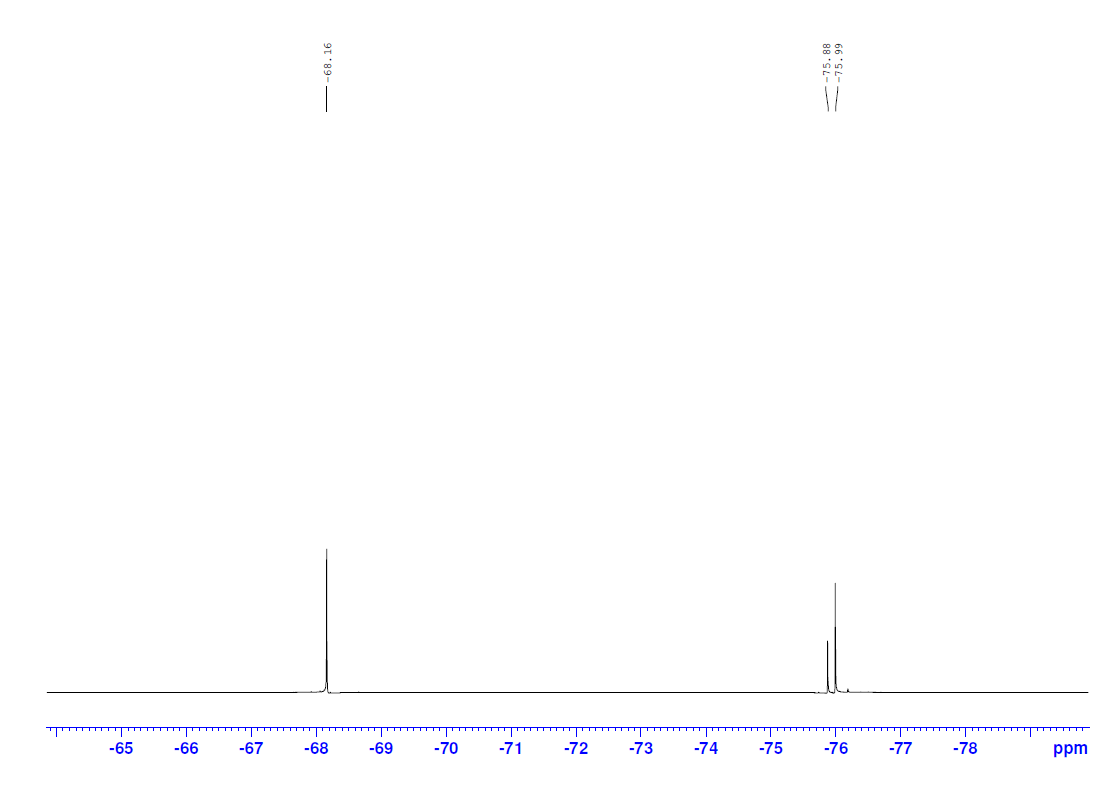


^1^H NMR, ^13^C NMR and ^19^F NMR spectra of compound **5** (400 MHz, CDCl_3_)

|   (with some solvent residue) |  |
| --- | --- |


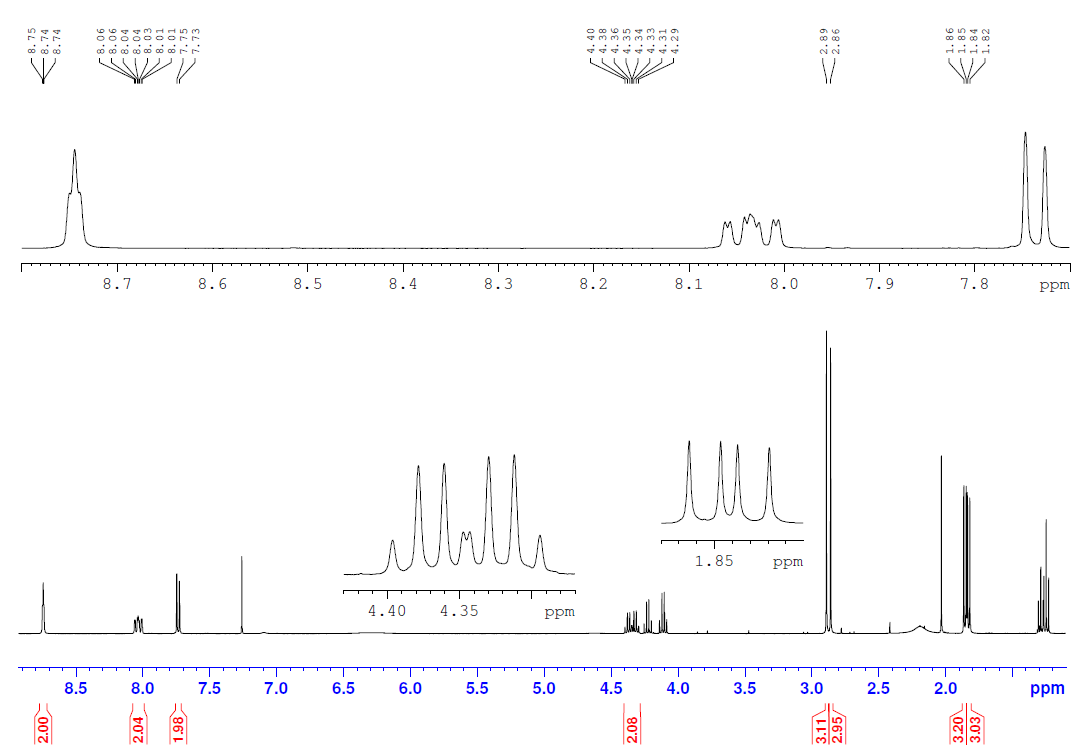


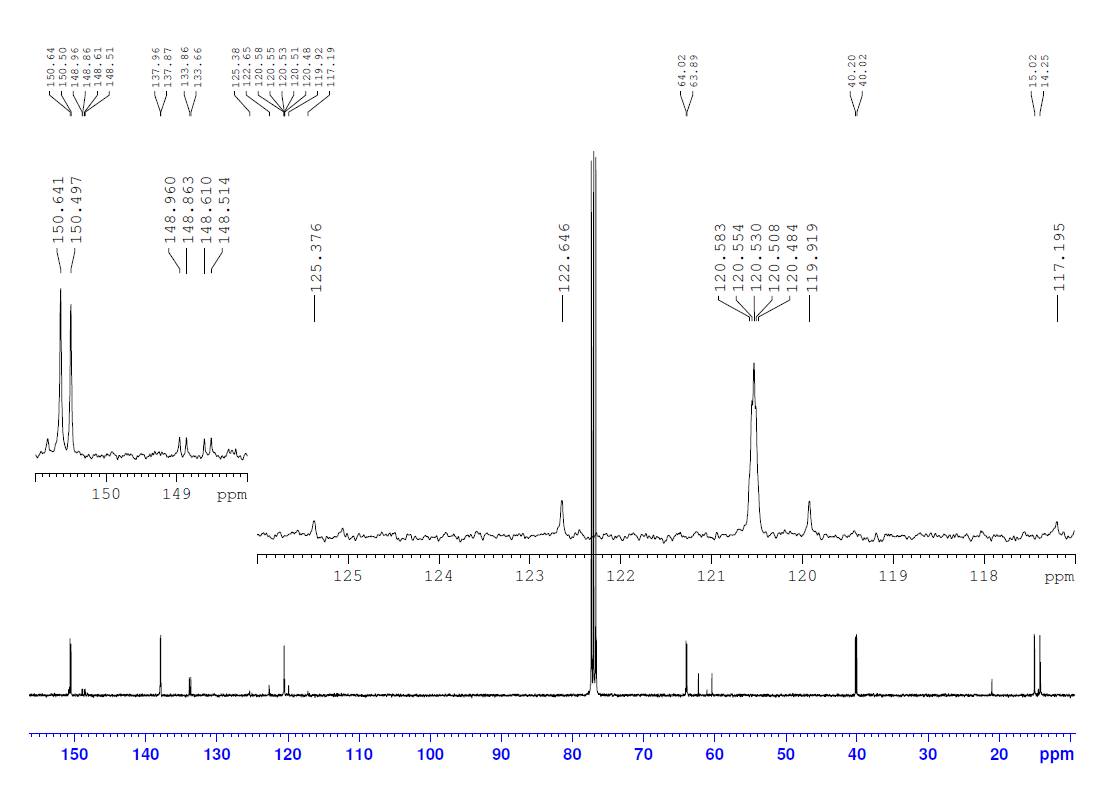


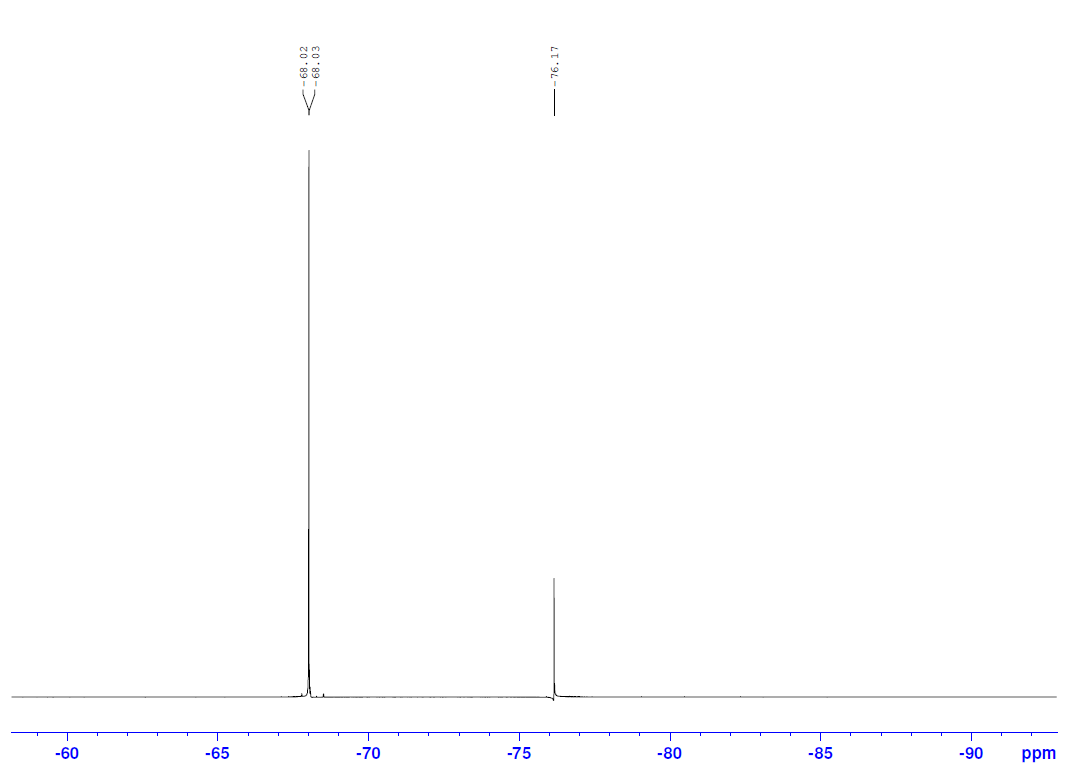


^1^H NMR, ^13^C NMR and ^19^F NMR spectra of SULFOXAFLOR **6** (400 MHz, CDCl_3_)

|  |  |
| --- | --- |


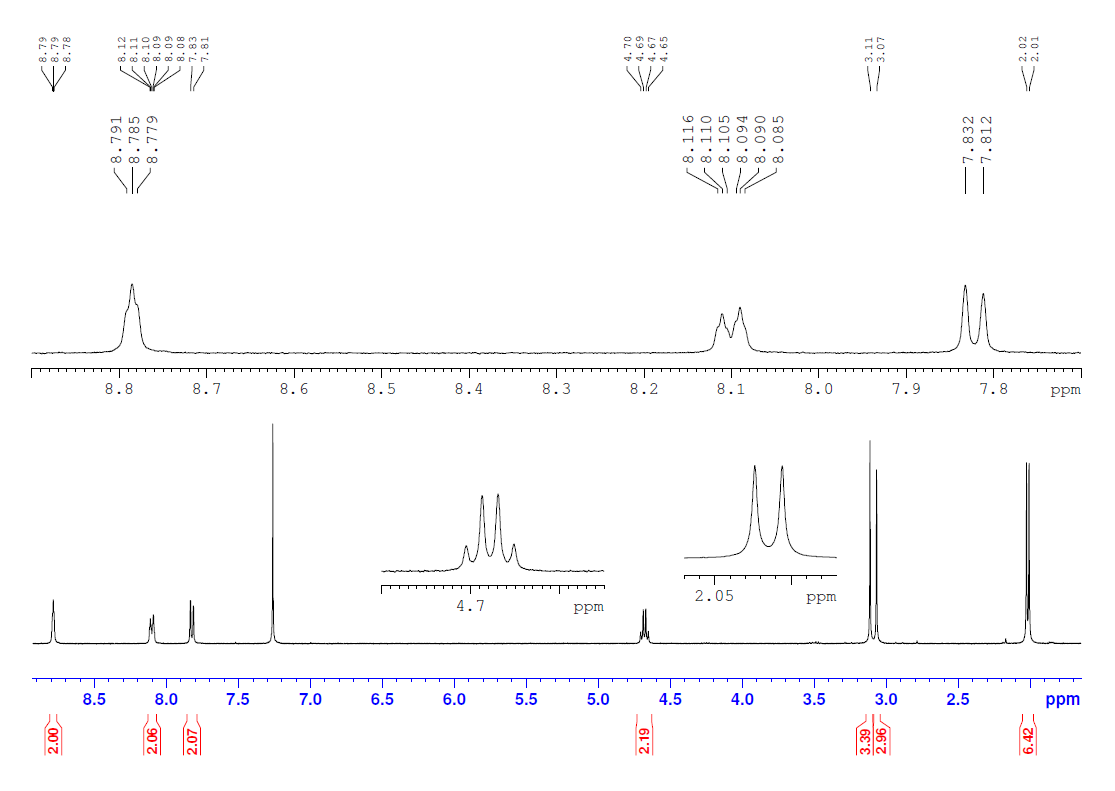


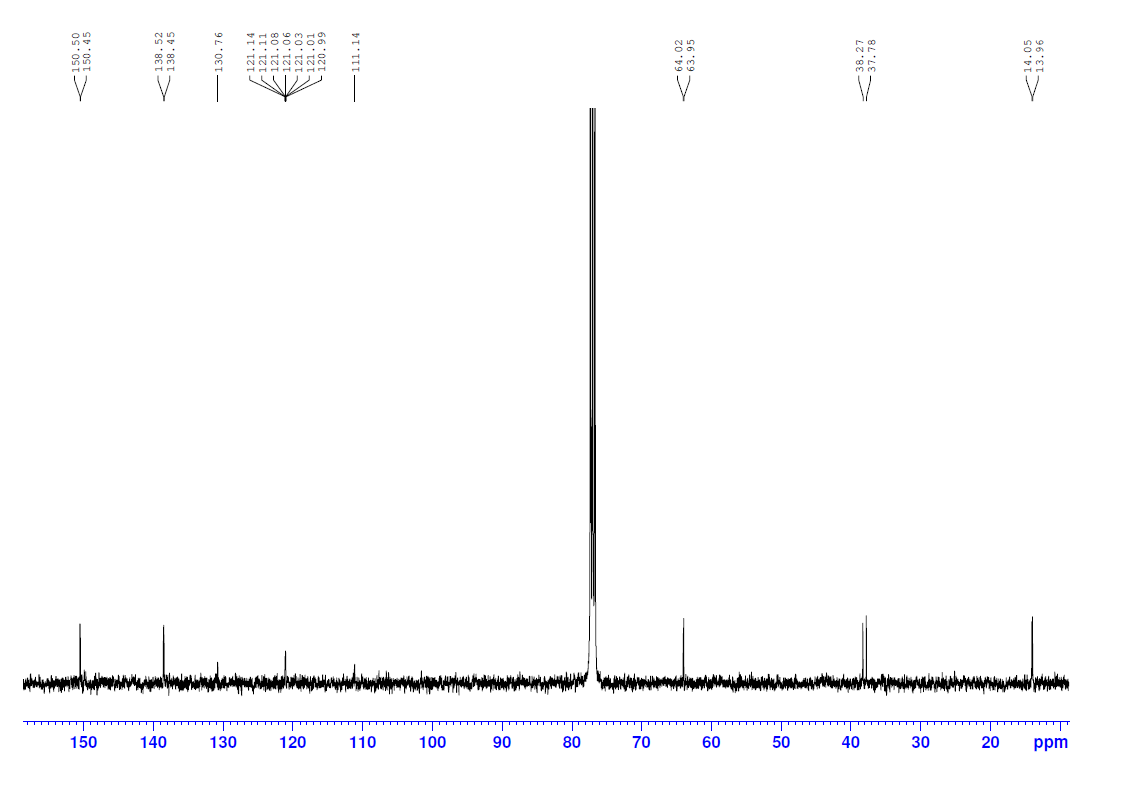


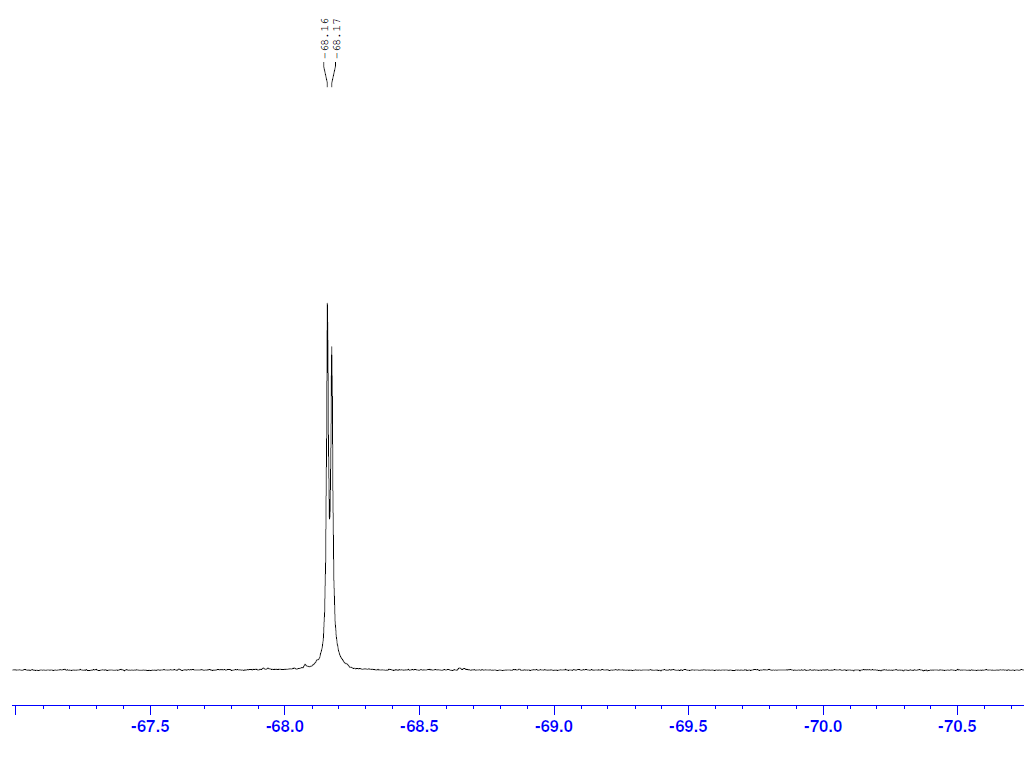


1. Arndt K.E. *et al*. Development of a Scalable Process for the Crop Protection Agent Isoclast. *Org. Process Res. Dev.* **2015**, 19, 454−462. DOI: 10.1021/acs.oprd.5b00007. [↑](#footnote-ref-1)
2. Cutler P. *et al*, Investigating the mode of action of sulfoxaflor: a fourth-generation neonicotinoid. *Pest Manag Sci* **2013**; *69*, 607–619. DOI 10.1002/ps.3413 [↑](#footnote-ref-2)
