# Supplementary material for: Impairments in learning and memory performances associated with nicotinic receptor expression in the honeybee Apis mellifera after exposure to a sublethal dose of sulfoxaflor
Source: PLoS One. 2022 Aug 3;17(8):e0272514. doi: 10.1371/journal.pone.0272514 (PMC9348702; doi:10.1371/journal.pone.0272514)
Supplement: S2 File — (DOCX) [file pone.0272514.s002.docx]

**Impairments in learning and memory performances associated with nicotinic receptor expression in the honeybee *Apis mellifera* after exposure to a sublethal dose of sulfoxaflor.**

Alison Cartereau^1^, Xavier Pineau^1^, Jacques Lebreton^2^, Monique Mathé-Allainmat^2^, Emiliane Taillebois^1^, Steeve H. Thany^1*^

^1^Université d’Orléans, Laboratoire de Biologie des Ligneux et des Grandes Cultures (LBLGC) USC INRAE 1328, 1 rue de Chartres, 45067 Orléans, France.

^2^ Nantes Université, CEISAM UMR CNRS 6230, UFR des Sciences et des Techniques, 2 rue de la Houssinière, BP 92208, 44322 Nantes, France.

**Fig 3B. SI. Script statistic test : Effect of sulfoxaflor sublethal dose on learning memory formation after exposure, 30 min before conditioning**

library(MASS)

library(readr)

library(lme4)
library(lmtest)

mod2 = glmer(response ~ trial*modalite+n+(1|id) + (1|session), family=binomial)

mod2

mod3 = glmer(response ~ trial*modalite+(1|id) + (1|session), family=binomial)

mod3

mod4 = glmer(response ~ trial+modalite+(1|id) + (1|session), family=binomial)

mod4

mod5 = glmer(response ~ modalite+(1|id) + (1|session), family=binomial)

mod5

mod6 = glmer(response ~ trial+(1|id) + (1|session), family=binomial)

mod6

mod7 = glmer(response ~ trial+n+(1|id) + (1|session), family=binomial)

mod7

X <- sum(residuals(mod4, type = "pearson")^2)

ddl <- df.residual(mod4)

1 - pchisq(X,ddl)

lrtest(mod4,mod6)

anova(mod4, mod6)

summary(mod4)

**Fig 4B. SI. Script statistic test : Effect of sulfoxaflor sublethal dose on learning memory formation after exposure 12 h before conditioning**

library(MASS)

library(readr)

library(lme4)
library(lmtest)

mod = glmer(response ~ trial*modality+coliny+n+(1|id) + (1|session), family=binomial)

mod

mod1 = glmer(response ~ trial*modality+coliny+(1|id) + (1|session), family=binomial)

mod1

mod2 = glmer(response ~ trial*modality+n+(1|id) + (1|session), family=binomial)

mod2

mod3 = glmer(response ~ trial*modality+(1|id) + (1|session), family=binomial)

mod3

mod4 = glmer(response ~ trial+modality+(1|id) + (1|session), family=binomial)

mod4

mod5 = glmer(response ~ modality+(1|id) + (1|session), family=binomial)

mod5

mod6 = glmer(response ~ trial+(1|id) + (1|session), family=binomial)

mod6

mod7 = glmer(response ~ trial+n+(1|id) + (1|session), family=binomial)

mod7

X <- sum(residuals(mod2, type = "pearson")^2)

ddl <- df.residual(mod2)

1 - pchisq(X,ddl)

anova(mod2,mod6)

**Fig 5B. SI. Script statistic test : Effect of sulfoxaflor sublethal dose on memory processes after exposure 3 h 30 after** **conditioning**.

library(MASS)

library(readr)

library(lme4)
library(lmtest)

mod2 = glmer(response ~ trial*modalite+n+(1|id) + (1|session), family=binomial)

mod2

mod3 = glmer(response ~ trial*modalite+(1|id) + (1|session), family=binomial)

mod3

mod4 = glmer(response ~ trial+modalite+(1|id) + (1|session), family=binomial)

mod4

mod5 = glmer(response ~ modalite+(1|id) + (1|session), family=binomial)

mod5

mod6 = glmer(response ~ trial+(1|id) + (1|session), family=binomial)

mod6

mod7 = glmer(response ~ trial+n+(1|id) + (1|session), family=binomial)

mod7

X <- sum(residuals(mod2, type = "pearson")^2)

ddl <- df.residual(mod2)

1 - pchisq(X,ddl)

anova(mod2,mod7)

**Fig 6B. SI. Script statistic test : Effect of sulfoxaflor sublethal dose on memory processes after exposure 23 h 30 after** **conditioning**.

library(MASS)

library(readr)

library(lme4)
library(lmtest)

mod2 = glmer(response ~ trial*modalite+n+(1|id) + (1|session), family=binomial)

mod2

mod3 = glmer(response ~ trial*modalite+(1|id) + (1|session), family=binomial)

mod3

mod4 = glmer(response ~ trial+modalite+(1|id) + (1|session), family=binomial)

mod4

mod5 = glmer(response ~ modalite+(1|id) + (1|session), family=binomial)

mod5

mod6 = glmer(response ~ trial+(1|id) + (1|session), family=binomial)

mod6

mod7 = glmer(response ~ trial+n+(1|id) + (1|session), family=binomial)

mod7

X <- sum(residuals(mod2, type = "pearson")^2)

ddl <- df.residual(mod2)

1 - pchisq(X,ddl)

anova(mod2,mod7)
